# ACNNT3: Attention-CNN Framework for Prediction of Sequence-Based Bacterial Type III Secreted Effectors

**DOI:** 10.1155/2020/3974598

**Published:** 2020-04-03

**Authors:** Jie Li, Zhong Li, Jiesi Luo, Yuhua Yao

**Affiliations:** ^1^School of Information Science and Technology, Zhejiang Sci-Tech University, Hangzhou 310018, China; ^2^School of Science, Zhejiang Sci-Tech University, Hangzhou 310018, China; ^3^Key Laboratory for Aging and Regenerative Medicine, Department of Pharmacology, School of Pharmacy, Southwest Medical University, Luzhou 646000, China; ^4^School of Mathematics and Statistics, Hainan Normal University, Haikou 571158, China

## Abstract

The type III secretion system (T3SS) is a special protein delivery system in Gram-negative bacteria which delivers T3SS-secreted effectors (T3SEs) to host cells causing pathological changes. Numerous experiments have verified that T3SEs play important roles in many biological activities and in host-pathogen interactions. Accurate identification of T3SEs is therefore essential to help understand the pathogenic mechanism of bacteria; however, many existing biological experimental methods are time-consuming and expensive. New deep-learning methods have recently been successfully applied to T3SE recognition, but improving the recognition accuracy of T3SEs is still a challenge. In this study, we developed a new deep-learning framework, ACNNT3, based on the attention mechanism. We converted 100 residues of the N-terminal of the protein sequence into a fusion feature vector of protein primary structure information (one-hot encoding) and position-specific scoring matrix (PSSM) which are used as the feature input of the network model. We then embedded the attention layer into CNN to learn the characteristic preferences of type III effector proteins, which can accurately classify any protein directly as either T3SEs or non-T3SEs. We found that the introduction of new protein features can improve the recognition accuracy of the model. Our method combines the advantages of CNN and the attention mechanism and is superior in many indicators when compared to other popular methods. Using the common independent dataset, our method is more accurate than the previous method, showing an improvement of 4.1-20.0%.

## 1. Introduction

Gram-negative bacteria can secrete proteins into host cells through a variety of secretion systems which affect the cell and its external environment. This process can be mediated by a variety of secretory systems, which can be divided into eight categories: type I to VIII secretory systems (T1SS-T8SS) [1]. Type I and III secretory systems are independent of signal sequences (sec), while types II and IV depend on signal sequences. The proteins secreted by the sec-dependent secretion system have a signal peptide sequence mainly composed of N-terminal hydrophobic amino acids which can guide the protein through the cell membrane. When the protein reaches the periplasm, the signal peptide is cut off. Type II and IV secretion systems remove the N-terminal part of the secreted protein in the periplasm. The difference between systems is that proteins pass through the outer membrane in different ways. When protein secreted by the type II secretion system passes through the outer membrane, an additional set of inner membrane and outer membrane proteins is needed to assist, while the type IV secretion system includes a series of autotransporters which form a hole in the outer membrane to make the protein pass through, autolyticly cut, and then release the protein. Neither the type I nor III secretion system processes the terminal amino acid of the secreted protein, nor does it appear that the secreted protein stays in the periplasm. The secretion signal of the protein secreted by the type I secretion system is located at about 60 amino acids from the C-terminal end of the protein. This secretory signal appears to be subfamily specific, and the secreted proteins are not easily cut by proteolytic enzymes. The type V secretion system is related to the secretion of macromolecular proteins and may also be sec-dependent.

The type III secretion system is a transmembrane channel formed by the multicomponent protein complex that has been widely encoded in many Gram-negative bacteria including Escherichia, Shigella, Yersinia, Salmonella, and Pseudomonas [2, 3]. It can change the signal transduction [4] and innate immune response [5] of host cells by secreting proteins or injecting these virulent proteins directly into host cells. Type III secretion systems (T3SSs) have been widely studied because they are critical for virulence in various human pathogens. There are many in vivo and in vitro methods for predicting T3SEs, and while some of them obtain good predictions, the experiments are complicated and time-consuming.

Some machine-learning methods have successfully been used to predict T3SEs, such as the Naïve Bayes (NB) [6], artificial neural network (ANN) [7], support vector machine (SVM) [8, 9], and random forest (RF) [10]. The disadvantage of these machine-learning methods is that features must be defined in advance, the appropriate selection of features will affect the prediction accuracy, and the flexibility of model change or update is limited [11]. Many deep-learning methods have been recently proposed, such as LSTM [12], ResNet [13], DenseNet [14], and VGG16 [15], and these methods can also be used in bioinformatics and other related fields [16, 17]. The deep-learning method DeepT3 [11] trained deep CNN using only one-hot encoding as the model feature input and achieved good prediction results in terms of accuracy. Since only one feature is input and CNN cannot connect the sequence context well when extracting sequence features, this method can be improved upon for predicting T3SEs.

The attention mechanism has recently gained popularity in neural networks because it can weigh the input features to measure the importance of each feature to the object recognition. It has widely been applied for text and image classification [18, 19], machine translation [20], and bioinformatics [21]. In this study, we propose a method for predicting T3SEs using N-terminal sequences based on the Attention-CNN. Our model extracts features of one-hot and PSSM from 100 residues of the N-terminal sequence and fuses them as the feature input. The attention layer in the model can well connect the front and back of the sequence, and the CNN module can well extract the features of the sequence. We combine these two modules to make the entire framework learn the features of the sequence to their maximum extent. The results show that our method is effective in predicting T3SEs; not only can it accurately capture protein transport target information, but it also performs better than the existing methods.

## 2. Materials and Methods

### 2.1. Dataset

We collected a comprehensive dataset from multiple bacterial species known as T3SEs and non-T3SEs from previous studies by Yang et al. [10], Wang et al. [22], and Tay et al. [23]. CD-HIT [24] with the sequence identity cutoff of 30% was used for sequence alignment to remove proteins with high similarity, and by skipping proteins with less than 100 amino acids, we obtained a balance dataset containing 283 T3 proteins and 311 non-T3 proteins.

We established our negative sample set by selecting type I to VIII secreted proteins of Gram-negative bacteria and removing type III secreted proteins and their homologues. In order to establish a 1 : 3 ratio of positive to negative [11], we randomly selected negative samples from the previous work of Dong et al. [8] and eliminated protein sequences with high similarity, resulting in a total of 835 negative samples.

There are two test sets used to evaluate our method. The independent dataset collected from Li et al. [11] contains 35 type III effectors and 86 non-type III effectors. The other test dataset is from the plant pathogen *P. syringae*. 85 type III effectors and 14 non-type III effectors that were not included in all models were collected from Baltrus et al. [25].

At present, most tools are based on the full sequence information of proteins or only 100 C-terminal residues [26]. In previous studies, N-terminal residues have been shown to also provide targeted information for protein transport [27–29], and the target information of T3SEs is usually located in the 50-100 N-terminal residues in different bacteria [30, 31]. Therefore, we have only used the N-terminal sequences in all the following calculations.

### 2.2. Feature Extraction

The feature input of our model is the combination of one-hot encoding and the PSSM of a protein. Each sequence is transformed into a one-hot matrix with 100 rows and 20 columns and a PSSM matrix with 100 rows and 20 columns, which are integrated into a combination matrix with 200 rows and 20 columns as the feature input. The 20 columns in the one-hot matrix correspond to 20 amino acids. One-hot encoding solves the problem of the classifier not effectively processing attribute data and expands the features to a certain extent. However, compared to PSSM, one-hot encoding is weaker with regard to protein feature extraction. Here, the introduction of PSSM enables the network model to better learn the characteristic preference of proteins, because PSSM features consider the position weight, number, and other parameters of each amino acid in the protein. PSSM also considers evolutionary information, so even the same residue may generate different characteristics, and it can effectively extract information from amino acid sequences. We used the PSI-BLAST [32] search database from UniprotKB/Swiss-Prot [33] to obtain the PSSM of the target protein. The matrix is an *L* × 20 matrix, where *L* represents the total number of residues in the target protein's amino acid sequence. At the same time, we use 1, 2, 3,…, 20 to represent the individual characters of the ordered 20 basic amino acids and get the corresponding number of columns. In summary, *U*_*i*→*j*_^⊕^ indicates the possibility that the *i* position of the amino acid sequence of the target protein is encoded as the basic amino acid *j* during the evolution process.

(1) $$\begin{array}{c} \text{PSSM}=\left[\begin{array}{cc} \begin{array}{cc} U_{1\to 1}^{⊕} & U_{1\to 2}^{⊕}\\ U_{2\to 1}^{⊕} & U_{2\to 2}^{⊕} \end{array} & \begin{array}{cc} \cdots & U_{1\to 20}^{⊕}\\ \cdots & U_{2\to 20}^{⊕} \end{array}\\ \begin{array}{cc} \vdots & \vdots \\ U_{L\to 1}^{⊕} & U_{L\to 2}^{⊕} \end{array} & \begin{array}{cc} \vdots & \vdots \\ \cdots & U_{L\to 20}^{⊕} \end{array} \end{array}\right]. \end{array}$$

### 2.3. Overview of Attention-CNN Model

The traditional CNN model includes convolution, pooling, and full connection layers, and it can be used to extract the sequence features of proteins. However, the sequence of a protein is more like a piece of text composed of amino acids, and since, when one amino acid may be related to the amino acids around it or those even farther away, it is not enough to extract these features using only the CNN mechanism. We also need to consider the information before and after the protein sequence and the correlation between discontinuous amino acids. Intuitively, an amino acid or a segment of amino acids may have a great impact on the protein sequence, so we can set a higher weight to this or this part of amino acids and have thus introduced the attention layer into the network.

Attention is a network structure model layer based on encode-decode, which has achieved satisfying prediction results compared to other traditional models in many fields including machine translation, picture description, and speech recognition. This implementation of the attention mechanism retains the intermediate output results of the input sequence of an LSTM encoder, then trains a model to selectively learn these inputs and associate the output sequence with them when the model outputs.

We added the attention and full connection layers in parallel after the convolution and pool layers, so that the model can not only take advantage of the mechanism of attention to learn the front and back features of the sequence, but also use the advantages of CNN feature extraction.

Our framework, ACNNT3, first uses multiple convolution and pooling layers to automatically learn protein sequence features, then takes the output feature vector as the input of the attention layer to calculate the score showing whether the neural network pays attention to the sequence features of the location. We define the output after convolution and pooling as a matrix *M*^*c*^(*d* × *q*), where *d* is the number of convolution kernels, *q* is the whole position after sequence pooling, and the column *j* of the feature map matrix *M*^*c*^ can be viewed as a feature vector (denoted by *V*_*j*_). *W*_*j*_ is the normalized importance score that is used to further average the columns of the feature map matrix *M*^*c*^. The dense matrix output through the attention network is *M*^*a*^, i.e.,

(2) $$\begin{array}{c} M^{a}=\overset{q}{\underset{j=1}{\sum }}w_{j}v_{j}, \end{array}$$

(3) $$\begin{array}{c} w_{j}=\frac{\exp\left(e_{j}\right)}{\sum_{j=1}^{q}\exp\left(e_{t}\right)}, \end{array}$$

where *e*_*j*_ is the importance score of the shared network and *W*_*j*_ is the relevant standardization score.

In order to integrate the features after convolution-pooling and the feature output by the attention layer, we first connect all the values in *M*^*c*^ and project them linearly to a value that represents the contribution of the whole sequence, represented by *S*^*c*^, then we concatenate it with the dense representation *M*^*a*^ and input it into the logistic regression classifier to obtain the prediction score, namely,

(4) $$\begin{array}{c} \text{Pred}\left(s\right)=\text{sigm}\left(\text{concat}\left(M^{a},S^{c}\right)\right), \end{array}$$

where *s* represents a position in the integrated sequence.

(5) $$\begin{array}{c} \;S^{c}=\text{dense}\left(\text{pool}\left(\text{conv}\left(\text{encode}\left(s\right)\right)\right)\right), \end{array}$$

where encode(.), conv(.), pool(.), concat(.), dense(.), and sigmoid(.) represent the unification of one-hot and PSSM encoding, convolution, maximum pooling, concatenation, dense connection, and sigmoid operation, respectively. At the same time, for a specific sequence, we can also output a weight vector, i.e.,

(6) $$\begin{array}{c} \text{AttMap}\left(s\right)=\left(w_{1},\cdots ,w_{q}\right). \end{array}$$

This formula is used to express the attention of the model to each position of the input sequence.

### 2.4. Model Training

**Figure 1:**
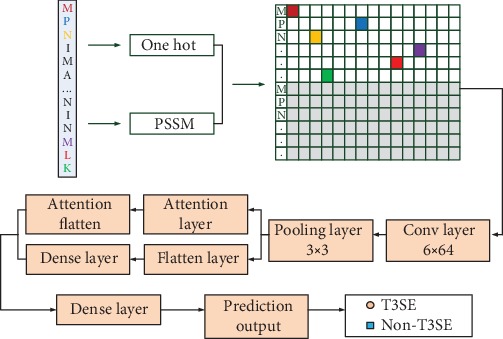
ACNNT3 architecture for T3SE prediction. Firstly, 64 1D convolution kernels with a length of 6 are convoluted to generate a 195 × 64 feature map, and then a 65 × 64 feature map is obtained through a 3 × 1 maximum pooling layer. The feature map is then input to the attention and full connection layers, and the two output results are combined to get 66 nodes. Finally, the 66 nodes are fully connected to the two output nodes, and the sigmoid function is used to activate to get the prediction probability of T3SE and non-T3SE.

**Figure 2:**
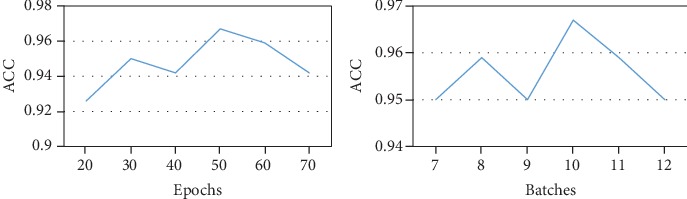
ACC comparison on the independent dataset under different epochs and batches.

ACNNT3 is composed of a series of modules which use the fusion features of 100 amino acids at the N-terminal of the protein as input to predict T3SEs (Figure 1). The ACNNT3 model consists of convolution, pooling, attention, and fully connected layers. We use crossvalidation to train our model and improve the generalization ability. The loss function uses a binary cross entropy loss function, and the optimizer uses the Adam algorithm. In Figure 2, we give the accuracy (ACC) comparison on the independent datasets under different epochs and batches. Since the dataset is not very large, the number of training epochs is set as 50 and the best batch value on the crossvalidation set is 10 as the optimal setting.

### 2.5. Performance Evaluation

We used 5-fold crossvalidation to estimate the classification performance of our model. Namely, we repeated the process five times and recorded the training parameters and average performance parameters for each time. The commonly used evaluation indexes for two-class classification are precision (PRE), sensitivity (SN), specificity (SP), F1 score, accuracy (ACC), and Matthew's correlation coefficient (MCC):

(7) $$\begin{array}{c} \text{PRE}=\frac{\text{TP}}{\text{TP}+\text{FP}}, \end{array}$$

(8) $$\begin{array}{c} \text{SN}=\frac{\text{TP}}{\text{TP}+\text{FN}}, \end{array}$$

(9) $$\begin{array}{c} \text{SP}=\frac{\text{TN}}{\text{TN}+\text{FP}}, \end{array}$$

(10) $$\begin{array}{c} \text{F}1\text{ score}=2\times \frac{\text{TP}}{2\text{TP}+\text{FP}+\text{FN}}, \end{array}$$

(11) $$\begin{array}{c} \text{ACC}=\frac{\text{TP}+\text{TN}}{\text{TP}+\text{FP}+\text{TN}+\text{FN}}, \end{array}$$

(12) $$\begin{array}{c} \text{MCC}=\frac{\left(\text{TP}\times \text{TN}\right)-\left(\text{FN}\times \text{FP}\right)}{\sqrt{\left(\text{TP}+\text{FN}\right)\times \left(\text{TN}+\text{FP}\right)\times \left(\text{TP}+\text{FP}\right)\times \left(\text{TN}+\text{FN}\right)}}, \end{array}$$

where TP, TN, FP, and FN represent the number of true positive, true negative, false positive, and false negative protein datasets, respectively.

The ROC curve is the relationship between the true positive and false positive rates, which is used to measure the comprehensive performance of different methods. The area under the ROC curve (AUC) is commonly used as a summary measure of diagnostic accuracy. In the ROC curve, the horizontal axis is the FPR (false positive rate, i.e., the ratio of wrongly predicted pairs over the total number of negative pairs), and the vertical axis is the TPR (true positive rate, i.e., the ratio of correctly predicted pairs over the total number of positive pairs). The maximum AUC is 1, which means a perfect prediction, and the AUC obtained by a random guess is 0.5.

## 3. Results

**Figure 3:**
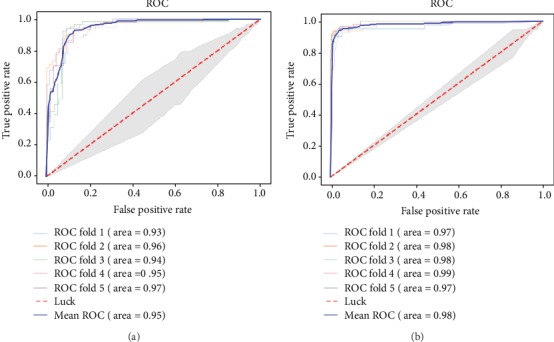
ROC curves on different training sets. (a) Use 5-fold crossvalidation experiment on training set 1. (b) Use 5-fold crossvalidation experiment on training set 2.

We have constructed a new prediction model to identify T3SEs by using a neural network that combines attention with CNN. In order to study the influence of the negative sample set on performance, we divided the training set into two parts. The positive to negative ratio of training set 1 is 1 : 1, and the positive to negative ratio of training set 2 is 1 : 3. The ACNNT3 model was trained using training sets 1 and 2, respectively. To evaluate the classification performance of our ACNNT3 model, we use ROC and AUC as the evaluation criterion. The ROC charts of 5-fold crossvalidation curves under training sets 1 and 2 are shown in Figures 3(a) and 3(b). We can see that the ACNNT3 model achieved a good performance on the ROC chart. The mean AUC of the model is 0.95 on training set 1 and 0.98 on training set 2. These results show that our ACNNT3 model can accurately classify T3SEs and non-T3SEs on both training sets.

### 3.1. Comparison of Different Features on the Same Network

**Figure 4:**
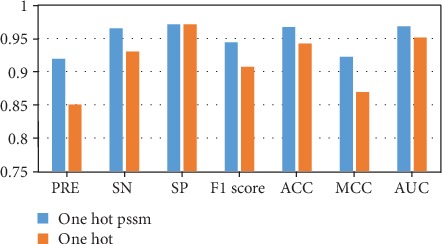
Comparison of experimental results of fusion feature and single feature under the same network model.

We take the one-hot single feature and the fused feature containing the one-hot matrix and PSSM as inputs, respectively, using ACNNT3 as the training model, and use the independent dataset to evaluate the two models. The results show that in all evaluation indexes, the model with the fusion feature is superior to the one with single feature training, thus verifying the proposed fusion feature's effectiveness (Figure 4). Compared to the one-hot single feature, the fusion feature is more comprehensive for the extraction of protein sequence information, and it can be seen from the experimental results that two types of features have good compatibility with each other.

### 3.2. Comparison of Different Deep-Learning Methods

We compared the results from different popular network models using the independent dataset with the same feature input, as shown in Table 1. For a class of sequential processing problems, the addition of an attention layer makes the network model strengthen the connection before and after the amino acid and the attention of important information in the sequence. From the experimental results, it can be seen that our network model ACNNT3 is better than the existing deep-learning framework for predicting T3SEs in many indicators.

### 3.3. Comparison with Existing Methods

**Figure 5:**
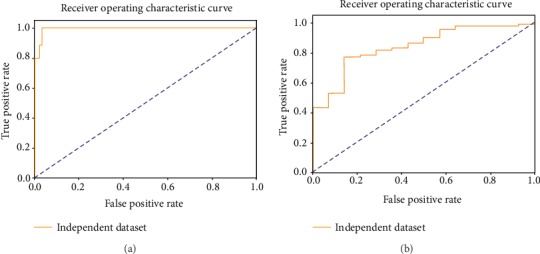
ROC curve of the best model selected from the 5-fold crossvalidation on two datasets. (a) ROC curve on a common independent dataset. (b) ROC curve on a *P. syringae* dataset.

In order to evaluate the effectiveness of our method, we compared the ACNNT3 performance with four popular methods, DeepT3 [11], BPBAac [22], Effective T3 [6], and BEAN2 [34], on the same independent dataset. The parameter settings of these methods are the same as those used by Li et al. [11]. We found that our ACNNT3-1 model has a higher SN, F1 score, ACC, and MCC than the other four methods (Table 2). The results also show that our method achieved satisfactory performance in almost all indicators. For the important index of ACC, the accuracy of ACNNT3-1 is 0.967, which is 9.9%, 4.1%, 15.7%, 20.0%, 9.6%, and 10.5% higher than ACNNT3-2, DeepT3-1, DeepT3-2, Effective T3, BPBAac, and BEAN2, respectively. In another *P. syringae* dataset, our model still performed better than the existing methods on the index of ACC (Table 3). The accuracy of ACNNT3-1 is 0.887. We selected the best model in the fivefold crossvalidation and used the independent and *P. syringae* datasets to test it. We also obtained the ROC curves of the model on two test sets (Figure 5). Overall, our method has been shown to be superior to all the latest methods in T3SE prediction and is reliably stable.

## 4. Conclusion

We have proposed a new prediction model for Gram-negative bacteria type III secreted proteins based on a deep neural network. In order to better learn the feature preference of type III secreted proteins, we integrated the one-hot encoding and PSSM extracted from the protein primary sequence as the feature input and embedded the attention layer into CNN to improve the model's prediction ability. This method outperforms other existing methods on most indicators, and using feature and network model comparisons, we have shown its advantages. In comparison with other popular methods, ACNNT3 is more accurate at predicting and recognizing T3SEs in the independent test set, which reflects its advantages and effectiveness. However, we found that ACNNT3's performance using the *P. syringae* dataset is not particularly obvious and was only slightly higher than the previous methods in terms of ACC and MCC. Our work in the future will focus on achieving better results in other experimental indicators and on applying this model for prediction using other large-scale datasets.

For easy implementation, all data used in this work and the source code for feature computing can be accessible at https://github.com/Lijiesky/ACNNT3.

## Figures and Tables

**Table 1:** Comparison with mainstream deep-learning methods.

Method	PRE	F1 score	ACC	MCC	AUC
ACNNT3	**0.919**	**0.944**	**0.967**	**0.922**	**0.968**
DenseNet	0.850	0.907	0.942	0.870	0.951
VGG16	0.846	0.892	0.934	0.847	0.937
ResNet	0.609	0.691	0.838	0.552	0.795
CNN	0.780	0.842	0.901	0.776	0.904
LSTM	0.875	0.933	0.959	0.909	0.961

The bold values indicate the best prediction results.

**Table 2:** Comparison of ACNNT3 and DeepT3, Effective T3, BPBAac, and BEAN2 on an independent dataset.

Method	PRE	SN	SP	F1 score	ACC	MCC	AUC
ACNNT3-1	0.919	**0.971**	0.965	**0.944**	**0.967**	**0.922**	0.968
ACNNT3-2	0.711	0.914	0.849	0.800	0.868	0.716	0.882
DeepT3-1	0.825	0.943	0.919	0.880	0.926	0.830	**0.974**
DeepT3-2	0.643	0.771	0.825	0.701	0.810	0.569	0.896
Effective T3	0.542	0.839	0.741	0.658	0.767	0.521	0.803
BPBAac	**0.944**	0.548	**0.988**	0.694	0.871	0.656	0.902
BEAN2	0.674	0.935	0.835	0.784	0.862	0.706	0.865

The bold values indicate the best prediction results.

**Table 3:** Comparison of ACNNT3 and DeepT3, Effective T3, BPBAac, and BEAN2 on a *P. syringae* dataset.

Method	PRE	SN	SP	F1 score	ACC	MCC	AUC
ACNNT3-1	0.900	0.976	0.357	0.936	**0.887**	0.452	0.667
ACNNT3-2	0.872	**0.988**	0.143	0.926	0.866	0.265	0.565
DeepT3-1	0.905	0.962	0.429	0.932	0.884	**0.472**	**0.838**
DeepT3-2	**0.913**	0.924	0.500	0.918	0.860	0.437	0.763
Effective T3	0.906	0.906	0.428	0.906	0.838	0.334	0.810
BPBAac	0.875	0.494	**0.571**	0.631	0.505	0.046	0.562
BEAN2	0.883	0.988	0.083	**0.938**	0.884	0.271	0.607

The bold values indicate the best prediction results.

## Data Availability

The data used to support the findings of this study are included within the article.
